# Assessing Physicians’ Knowledge, Attitudes, and Practices in Occupational Disease Diagnosis and History Taking

**DOI:** 10.3390/ijerph22111763

**Published:** 2025-11-20

**Authors:** Salim Al-Busaidi, Nasiba Al Maqrashi, Sheikha Alwahshi, Yaqoub Alsaidi

**Affiliations:** 1 Department of Medicine, Sultan Qaboos University Hospital, University Medical City, Affiliated with Sultan Qaboos University, Muscat 123, Oman; salim.sas1992@gmail.com; 2 Internal Medicine Residency Program, Oman Medical Specialty Board, Muscat 132, Oman; nasiba.almaqrashi@gmail.com; 3 Family Medicine Residency Program, Oman Medical Specialty Board, Muscat 132, Oman; sheikhasaif77@gmail.com; 4 Department of Family Medicine and Public Health, Sultan Qaboos University Hospital, University Medical City, Affiliated Sultan Qaboos University, Muscat 123, Oman

**Keywords:** occupational disease, occupational health, health knowledge, attitudes, practice

## Abstract

**Background and Aim:** Oman’s rapid industrial development has resulted in changing occupational exposures, emphasizing the importance of enhancing physicians’ proficiency in occupational history-taking and disease identification. This study sought to evaluate physicians’ knowledge, attitudes, and practices (KAP) in diagnosing occupational diseases and collecting occupational histories, while also identifying perceived barriers to effective recognition and reporting. **Method:** Data were collected utilizing a structured, self-administered questionnaire adapted from a validated instrument that assesses physicians’ knowledge, attitudes, and practices (KAP) concerning occupational diseases. The survey was conducted among internal medicine and family medicine physicians at a tertiary academic hospital. **Result:** Of 252 eligible physicians, 146 (57.9%) responded. Moderate levels were most common for knowledge (45.2%) and attitude (65.1%), while practice was most frequently high (45.9%). Prior training strongly predicted high knowledge (aOR = 7.23, 95% CI: 2.99–17.49; *p* < 0.001). Family Medicine physicians were more likely to achieve high knowledge (aOR = 2.42, 95% CI: 1.03–5.72; *p* = 0.043) but less likely to report high attitude scores (aOR = 0.32, 95% CI: 0.14–0.76; *p* = 0.010). Training also showed non-significant trends toward improved attitude and practice. **Conclusions:** Occupational health training is strongly linked to improved knowledge, with indications of benefits for attitude and practice. Specialty differences highlight the need to integrate occupational medicine into curricula and strengthen institutional support to enhance recognition of occupational diseases.

## 1. Introduction

Occupational diseases represent a significant and underrecognized public health burden worldwide. According to global estimates, over 2 million deaths annually are attributable to occupational exposures, with nonfatal occupational diseases affecting over 160 million individuals each year [1,2,3]. In Oman, these conditions remain underreported in official statistics, largely due to underdiagnosis and misidentification. As a result, the number of reported cases from Oman and across the region does not reflect the true burden observed internationally [2]. This is largely due to gaps in physicians’ awareness, attitudes, and clinical practice related to occupational health [1,4].

In Oman, as in many countries with rapidly growing industrial sectors, occupational health systems are challenged by a shortage of occupational medicine specialists and insufficient preparedness among non-specialist physicians to identify and report work-related illnesses [2]. Non-occupational medicine physicians, particularly in primary care and specialty practices such as pulmonology, rheumatology, and internal medicine, play a central role in recognizing the link between occupational exposures and disease [2,4,5]. Yet, studies consistently show that many clinicians fail to systematically inquire about occupational history or consider occupational etiology in differential diagnoses [4,5,6]. For instance, in a large national study from Turkey, only 44.1% of family medicine physicians reported routinely obtaining detailed occupational histories, and most lacked formal education or training in the diagnosis of occupational diseases [6]. Similarly, research in Egypt found that insufficient knowledge, a lack of structured history-taking tools, and time constraints were significant barriers to proper occupational history-taking and occupational disease diagnosis [1].

Failing to diagnose occupational diseases extends beyond individual health outcomes, resulting in continued hazardous exposures for affected individuals and their coworkers, missed opportunities for compensation, and the absence of preventive workplace interventions [1,7,8]. Moreover, underreporting of occupational diseases skews public health surveillance data, masking the true burden of work-related illness and impeding targeted policy responses [2,9]. In this context, the role of physicians, globally, extends beyond clinical care to include public health advocacy and surveillance. Yet, in Oman, this role remains underdeveloped due to multiple systemic and educational barriers [2]. Studies from diverse international settings—including France, Turkey, and Egypt—have shown that physicians’ engagement with occupational disease diagnosis is strongly influenced by prior training in occupational health, familiarity with legal procedures, and availability of institutional support [1,4,6].

Given the growing complexity of work environments, the evolving landscape of occupational exposures, and the extreme shortage of trained occupational medicine physicians, there is an urgent need to strengthen physician competence in occupational history-taking and diagnosis [9,10]. This study aims to evaluate the current knowledge, attitudes, and practices of family and internal medicine physicians in diagnosing occupational diseases and obtaining occupational histories, while identifying the barriers that hinder effective recognition and reporting. Through this, we aim to inform educational and policy strategies that enhance physician engagement in occupational health.

## 2. Methods

### 2.1. Study Design, Setting, and Participants

This descriptive cross-sectional study was conducted at Sultan Qaboos University Hospital (SQUH), a tertiary academic medical center in Muscat, Oman. The study targeted staff physicians affiliated with the Departments of Internal Medicine and Family Medicine, as well as residents enrolled in training programs for Internal Medicine and Family Medicine. The residents included had structured postgraduate training and substantial clinical exposure across diverse healthcare settings, including major hospitals and primary care services throughout Oman. Inclusion criteria were physicians working in internal medicine or family medicine departments at the study institution during the recruitment period. Exclusion criteria included physicians from other specialties and those who declined to participate. Data collection was conducted between March and May 2025. The sample size was calculated based on a previously reported low knowledge prevalence of 20% among physicians regarding occupational diseases [1]. Using a 5% margin of error and a 95% confidence level, and after applying the finite population correction for the total eligible population of 252, the minimum required sample size was calculated to be 139 participants, accounting for a 10% expected nonresponse rate. Ethical approval for this study was obtained from the Medical Research Ethics Committee (MREC) of the College of Medicine and Health Sciences, Sultan Qaboos University (MREC #3516, Ref. No. SQU-EC/021/2025).

### 2.2. Study Tool and Data Collection

We collected data using a structured, self-administered questionnaire adapted from a validated instrument developed by Sehsah et al. [1], which assessed physicians’ knowledge, attitudes, and practices related to occupational diseases and occupational history taking. Permission to use and adapt the tool was obtained from the original authors. The original instrument demonstrated high content validity (I-CVI: 0.9–1.0) and internal consistency (Cronbach’s alpha = 0.87). To ensure the tool was relevant to the Omani context, minor modifications were made, including adjustments to terminology and the addition of local and regional occupational disease examples, such as those prevalent in industries like oil and gas, construction, and manufacturing in Oman. Furthermore, the questionnaire was tailored to reflect the Omani healthcare system, including the local compensation framework and occupational disease reporting practices. The adapted version of the questionnaire was reviewed by members of the study team with expertise in occupational health to ensure both contextual accuracy and clarity. A pilot test was conducted among ten physicians (five internal medicine and five family medicine) who were not part of the main study. Their feedback focused on question clarity and contextual relevance, leading to minor wording refinements without altering item content. This process supported the content validity of the adapted questionnaire, while the construct validity was maintained by preserving the original domain structure of the Sehsah et al. instrument. The internal consistency of the adapted questionnaire was recalculated using Cronbach’s alpha, which yielded values ranging from 0.82 to 0.95, confirming good to excellent reliability.

The final questionnaire comprised four domains: knowledge (11 items), attitudes (4 items), practice (17 items), and barriers (unscored, descriptive). Domain scores were calculated, converted to percentages, and categorized as low (<60%), moderate (60–79.9%), or high (≥80%), based on thresholds commonly used in previous studies. A sensitivity analysis, including alternative thresholds (50%, 70%, 90%), and visual inspection of histograms confirmed that these thresholds were appropriate for our dataset.

A single-use questionnaire link was distributed electronically to the target population via Google Forms through institutional email groups and departmental communication channels. The questionnaire was administered in English, which is the standard language of professional communication among physicians in Oman. The study employed a convenience sampling approach, whereby all eligible physicians were invited to participate voluntarily. Participation was voluntary and anonymous, and informed consent was obtained electronically before participation.

### 2.3. Statistical Analysis

Data were analyzed using Stata version 17.0 (StataCorp LLC, College Station, TX, USA). Descriptive statistics, including frequencies, percentages, means, and standard deviations, were used to summarize participant characteristics and domain scores. Scores for knowledge, attitude, and practice were calculated, expressed as percentages, and categorized as low (<60%), moderate (60–79.9%), or high (≥80%).

Inferential analyses included independent *t*-tests for two-group comparisons and one-way ANOVA with Bonferroni post hoc correction for comparisons across multiple groups. Bivariate analyses identified associations between participant characteristics and domain scores. Variables with *p* < 0.10 in bivariate analysis were entered into multivariable models. Effect sizes were calculated to complement significance testing, using Cohen’s *d* for mean comparisons, η^2^ for ANOVA, and McFadden’s pseudo-R^2^ for regression models.

We used multinomial logistic regression to assess predictors of categorical KAP levels (Low, Moderate, High) for knowledge, attitude, and practice. Odds ratios (ORs), 95% confidence intervals (CIs), and *p*-values were reported, with significance set at *p* < 0.05. line Collinearity was assessed using the Variance Inflation Factor (VIF), with all VIFs below 2, indicating no multicollinearity.

Barriers to taking occupational history were analyzed descriptively and summarized using frequencies and percentages.

## 3. Results

### 3.1. Participant Characteristics

Out of 252 eligible physicians, a total of 146 completed the survey, yielding a response rate of 57.9%. The majority of respondents were female 82.2% and Omani nationals 89%. Most participants were residents 78.1%, with the remainder comprising staff physicians at the specialist or consultant level. Participants were nearly equally distributed between the Family Medicine 52.7% and Internal Medicine 47.3% departments. Approximately 56.2% of the respondents had less than five years of clinical experience, whereas 27.4% had between five and ten years, and 16.4% had more than ten years of experience (Table 1).

### 3.2. Knowledge, Attitude, and Practice Scores

The mean knowledge score was 7.65 ± 1.62 out of 11, corresponding to 69.5%. Nearly half 45.2% achieved moderate knowledge scores, while 23.3% were categorized as having low knowledge and 31.5% as having high knowledge. The attitude domain showed a mean percentage score of 74.7 ± 15.1%, with most participants 65.1% demonstrating a moderate attitude, 26.7% a high attitude, and 8.2% a low attitude score. The practice domain yielded the highest mean percentage score among the three domains, at 78.8 ± 13.3%, with 45.9% of participants achieving high practice scores, 40.4% moderate, and 13.7% low scores.

### 3.3. Factors Associated with Knowledge, Attitude, and Practice

Bivariate analysis (Table 2) demonstrated several significant and suggestive associations between participant characteristics and levels of knowledge, attitude, and practice.

In the knowledge domain, higher scores were significantly associated with longer clinical experience (*p* = 0.015) and prior occupational health training (*p* < 0.001). Among those with high knowledge scores, 32.6% had over 10 years of experience, compared to just 2.9% among those with low scores. Likewise, 82.6% of participants with high knowledge had received prior training, compared to only 26.5% in the low category. A trend was also observed for professional position (*p* = 0.060), with consultants comprising 15.2% of those with high knowledge scores, but none among the low scorers.

In the attitude domain, participants from Internal Medicine were significantly more likely to report high attitude scores than those from Family Medicine (69.2% vs. 30.8%, *p* = 0.005). Prior training also showed a trend toward favorable attitude levels (*p* = 0.052), with 69.2% of those with high attitude scores having received training, compared to 33.3% in the unfavorable category.

**Figure 1:**
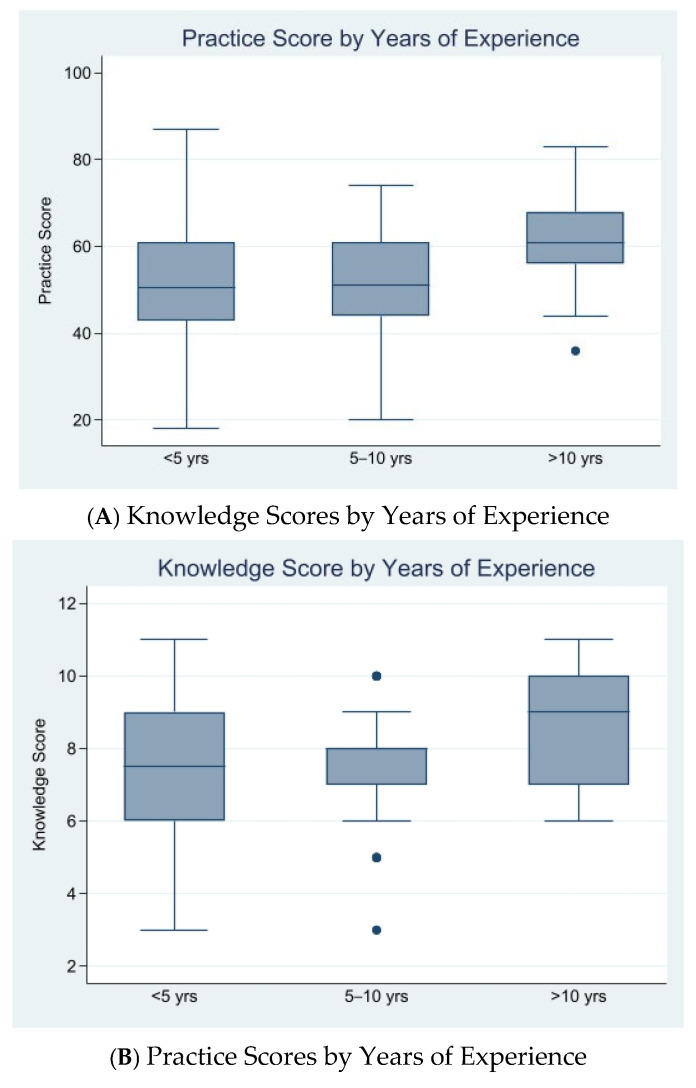

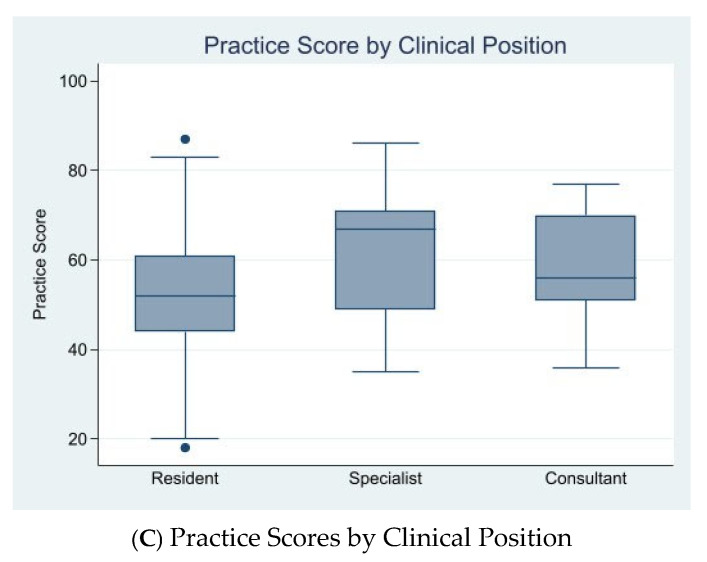
Knowledge and Practice Scores by Experience and Position.

In the practice domain, higher scores were significantly associated with clinical experience (*p* = 0.008). Specifically, 31.3% of participants with over 10 years of experience had high practice scores, compared to only 5.0% in the low category. A trend was also noted for prior training (*p* = 0.055), with 59.7% of those with high practice scores having received training, compared to 30.0% with no training. The trends in knowledge and practice scores across levels of clinical experience and professional position are presented in Figure 1.

Binary logistic regression analysis (Table 3) showed that prior training in occupational diseases was a strong independent predictor of high knowledge scores (aOR = 7.23, 95% CI: 2.99–17.49; *p* < 0.001). Physicians in Family Medicine were also more likely to achieve high knowledge scores compared with those in Internal Medicine (aOR = 2.42, 95% CI: 1.03–5.72; *p* = 0.043). In the attitude domain, Family Medicine physicians were less likely to report high attitude scores than Internal Medicine physicians (aOR = 0.32, 95% CI: 0.14–0.76; *p* = 0.010). No other predictors were significantly associated with high attitude or high practice levels.

### 3.4. Barriers to Occupational History Taking

**Figure 2:**
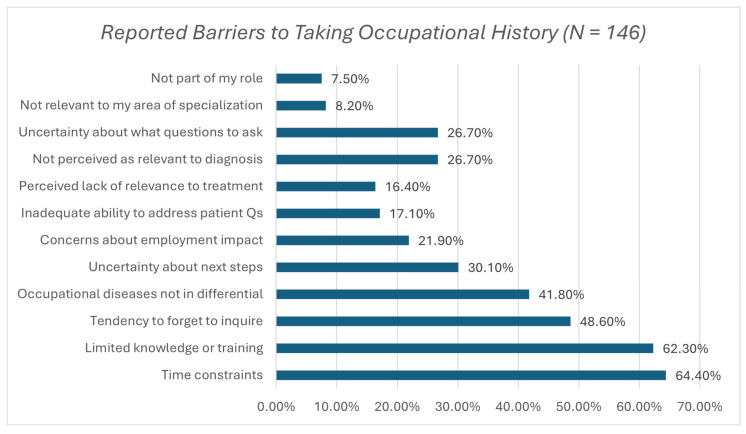
Reported barriers to obtaining a comprehensive occupational history or evaluating the potential for an occupational disease among physicians (N = 146). Note: “Percentages represent the proportion of respondents who identified each barrier (multiple responses allowed).”.

Participants reported a range of barriers that hinder the routine assessment of occupational history. The most commonly cited barriers were time constraints due to a busy schedule 64.4%, limited knowledge or training on occupational diseases 62.5%, and a tendency to overlook inquiring about occupational exposures 48.6%. Other reported barriers included uncertainty about the relevance to diagnosis 41.1%, perception that it does not apply to their specialty 27.4%, and lack of adequate training to address patient questions 17.1%. These are summarized in Figure 2.

## 4. Discussion

This study assessed physicians’ knowledge, attitudes, and practices regarding occupational disease diagnosis in Oman. Knowledge and attitude scores varied by training and specialty, while practice performance was moderate overall. Effect size measures indicated that the observed associations were small to moderate in magnitude, consistent with the cross-sectional nature of the study.

### 4.1. Impact of Experience and Training on Knowledge

The majority of participants possess a moderate to low level of knowledge, while nearly one-third demonstrate a high level of knowledge. Higher knowledge levels were observed among physicians with over a decade of experience and among those who had received prior occupational health training, indicating an association between these factors and enhanced awareness. Participants who had received prior training scored, on average, 1.38 points higher than those without such training (*p* < 0.001), and the odds of being in the high-knowledge category were over seven times greater (aOR 7.23, 95% CI: 2.99–17.49; *p* < 0.001), suggesting a strong statistical association between training exposure and knowledge level. Similar findings have been reported in previous studies from Europe and the Middle East, where occupational health training was consistently associated with increased physician awareness and diagnostic confidence regarding occupational diseases [1,8]. Family Medicine physicians were also more likely to achieve high knowledge compared to Internal Medicine physicians (aOR 2.42, 95% CI: 1.03–5.72; *p* = 0.043). This trend may reflect family medicine physicians’ broader community-oriented scope and greater emphasis on holistic patient assessment, which inherently includes social and occupational context. Such training frameworks may foster better recognition of workplace-related disease risk. Interestingly, this contrasts with findings from Gök et al. (2020), who assessed family medicine physicians in Turkey and reported generally limited knowledge regarding occupational diseases [8]. However, trained physicians in their study demonstrated greater knowledge and diagnostic confidence, which indicates that targeted education is likely correlated with greater competency within this specialty.

### 4.2. Specialty-Based Differences in Attitude Domains

Beyond knowledge, physicians’ attitudes play a critical role in determining how occupational health principles are applied in clinical settings. The majority of participants in this study reported favorable attitudes toward occupational disease evaluation compared to studies in Egypt and Turkey [1,6,11]. Moreover, this study shows no statistically significant difference in experience level or training, although trained physicians showed a non-significant trend toward higher attitude scores (*p* = 0.052). Internal Medicine physicians had significantly higher favorable attitude scores than Family Medicine physicians (aOR 0.32, 95% CI: 0.14–0.76; *p* = 0.010). This pattern might reflect the clinical realities faced by internists, who often manage complex, multisystem diseases requiring detailed history-taking and diagnostic reasoning. Such exposure could reinforce awareness of occupational determinants of health, though this interpretation remains hypothetical. It is also possible that the structured approach of family medicine physicians emphasizes preventive aspects more than diagnostic attribution, which may partly explain the attitudinal contrast observed between the two specialties. While our findings suggest specialty-related attitudinal variations, earlier studies did not demonstrate such differences, implying that contextual or institutional factors specific to Oman’s healthcare system may influence physicians’ orientation toward occupational health. Sehsah et al. (2024) [1] compared surgical and non-surgical physicians and found no significant differences in attitude scores. However, the study did not analyze differences between internal medicine and family medicine physicians specifically, which may explain the variation in findings [1].

### 4.3. Practice Performance and Its Association with Experience and Seniority

Nevertheless, positive attitudes do not always translate into consistent clinical practice, as reflected in the following domain. Overall, the practice scores are at a moderate and high level in this study (40.4 vs. 45.9, respectively). Longer clinical experience was significantly associated with higher practice scores in bivariate analysis (*p* = 0.008). However, this relationship was no longer significant after adjustment for other variables in the multivariable model (aOR 1.54, 95% CI 0.68–3.46; *p* = 0.297). This pattern suggests that while experience initially appears to influence occupational health practice, its effect is likely mediated by other factors such as training exposure and institutional context. These findings align with prior reports, showing that experiential learning contributes to occupational history-taking behavior but that sustained practice change requires supportive systems, feedback mechanisms, and ongoing education. Similar findings have been found that more experienced tended to demonstrate better adherence to occupational history-taking and reported greater diagnostic confidence [1]. Conversely, a study conducted over 2000 patient charts in a major U.S. teaching hospital found that occupational histories were documented in fewer than one-third of cases [12]. Another study in Thailand of 230 medical records found that only 45.7% of treating doctors considered recording patient occupation, and only 24.3% of records had the correct existing occupation when verified [13]. Another study showed that surgery students were less likely than internal medicine students to gather data about industry (41.6% vs. 66.6%, *p* < 0.001), and occupation (57.4% vs. 79.7%, *p* < 0.001) [14]. Their findings highlight a general underutilization of occupational history-taking in routine clinical practice, without specific comparisons across physician seniority levels.

### 4.4. Association of Training with Knowledge, Attitude, and Practice

Occupational health training was significantly associated with higher knowledge scores and showed non-significant trends toward higher attitude and practice scores. This association was statistically significant for knowledge (*p* < 0.001), while differences in attitude (*p* = 0.052) and practice (*p* = 0.055) did not reach significance. The relatively immediate and measurable gains in knowledge may reflect individual initiative and the comparative ease of acquiring factual information, whereas meaningful shifts in attitude and behavior often require sustained reinforcement, greater awareness of their importance, and supportive systems to enable change. The borderline findings for attitude and practice may reflect short or inconsistent training exposure and institutional barriers that limit the translation of knowledge into sustained changes in behavior. This highlights that education alone may be necessary but not sufficient for long-term behavioral change—underscoring the role of organizational policies and clinical reminders in translating knowledge into consistent practice.

### 4.5. Systemic Barriers to Effective Occupational History-Taking

The recurring barriers identified—time constraints, insufficient training, and low diagnostic prioritization—point to system-level challenges rather than individual physician shortcomings. These reflect structural pressures within high-volume clinics and limited institutional emphasis on occupational health. These findings are consistent with those of other research studies [1,15,16,17]. A study conducted in Egypt found that the most commonly reported barriers included insufficient knowledge about occupational diseases, lack of time with patients due to busy schedules, and difficulty identifying the origin of occupational illnesses [1]. Furthermore, another study reported that over 70% of U.S. physicians cited time constraints as the principal obstacle to recognizing occupational disease [16], while in the UAE, more than 50% of physicians identified time and workflow limitations as barriers to comprehensive patient history-taking [15]. Addressing these constraints may require embedding occupational history prompts in electronic medical records and integrating occupational health awareness into continuing professional development programs, consistent with international frameworks such as the *International Labour Organization (ILO) List of Occupational Diseases* [18].

### 4.6. Limitations

While this study provides valuable insights into the knowledge, attitudes, and practices of physicians, certain methodological constraints should be considered when interpreting these results. First, the cross-sectional design limits the ability to establish causality between variables. Second, reliance on self-reported data may have introduced bias, particularly in the assessment of clinical practices. Third, our sample predominantly comprised residents (78.1%) and female physicians (82.2%), which reflects the actual composition of the targeted cohort, where women form the majority [19,20]. While this reduces the risk of sampling error within our target group, it may limit generalizability to the wider physician workforce, including other specialties, senior physicians, and private-sector clinicians. The overlap between gender and specialty distribution should be considered when interpreting the findings, as the two specialties we evaluated in this study emphasize in preventive care and chronic disease management, which may provide fewer encounters with acute occupational injuries or industrial hazards compared to other specialties. Lastly, the use of a structured quantitative questionnaire may have restricted deeper exploration of contextual or institutional factors influencing practice. This being said, to enhance occupational KAP of current and future physicians in occupational diseases and the recognition of work-related diseases in clinical settings, the medical curriculum and training must be part of the medical student education through case-based learning and practical sessions. Mandating electronic templates, prompts, or checklists in the healthcare system may facilitate routine documentation of occupational exposures. Additionally, dedicated occupational health clinics should be established within hospital systems, and further qualitative and interventional studies should be conducted to explore barriers and evaluate the effectiveness of training.

## 5. Conclusions

This study found that physicians in Oman generally report moderate to reasonable levels of knowledge, attitudes, and practices concerning occupational health. Performance was significantly associated with prior occupational health training for knowledge, and with specialty for both knowledge and attitude. Non-significant but consistent trends were also observed toward improved attitude and practice among trained physicians. These findings highlight the need to embed occupational health principles in medical education and to strengthen institutional systems that support their application in clinical settings. Efforts to enhance physicians’ engagement with occupational health should prioritize structured education, user-friendly clinical tools, and sustained institutional support, approaches that align with the associative evidence demonstrated in this study.

## Figures and Tables

**Table 1:** Participant Demographic and Professional Characteristics (N = 146).

Characteristic	Frequency (n)	Percentage (%)
Sex		
Male	26	17.8%
Female	120	82.2%
Nationality		
Non-Omani	16	11.0%
Omani	130	89.0%
Specialty		
Internal Medicine	69	47.3%
Family Medicine	77	52.7%
Current Position		
Resident	114	78.1%
Specialist	22	15.1%
Consultant	10	6.9%
Years of Experience		
<5 years	82	56.2%
5–10 years	37	25.3%
>10 years	27	18.5%

**Table 2:** Association of Knowledge, Attitude, and Practice Levels with Participant Characteristics (N = 146).

Domain	Variable	Category	Low (% (n))	Moderate (% (n))	High (% (n))	*p*-Value
Knowledge	Total		23.3% (34)	45.2% (66)	31.5% (46)	
	*Sex*	*Male*	8.8% (3)	21.2% (14)	19.6% (9)	*0.287*
		*Female*	91.2% (31)	78.8% (52)	80.4% (37)	
	*Specialty*	*Internal Medicine*	55.9% (19)	48.5% (32)	39.1% (18)	*0.321*
		*Family Medicine*	44.1% (15)	51.5% (34)	60.9% (28)	
	*Position*	*Resident*	88.2% (30)	77.3% (51)	71.7% (33)	*0.06*
		*Specialist*	11.8% (4)	18.2% (12)	13.0% (6)	
		*Consultant*	0.0% (0)	4.6% (3)	15.2% (7)	
	*Experience*	*<5 years*	70.6% (24)	54.6% (36)	47.8% (22)	***0.015***
		*5–10 years*	26.5% (9)	28.8% (19)	19.6% (9)	
		*>10 years*	2.9% (1)	16.7% (11)	32.6% (15)	
	*Training*	*No*	73.5% (25)	50.0% (33)	17.4% (8)	**<0.001**
		*Yes*	26.5% (9)	50.0% (33)	82.6% (38)	
Attitude	Total		8.2% (12)	65.1% (95)	26.7% (39)	
	*Sex*	*Male*	16.7% (2)	17.9% (17)	17.9% (7)	***0.994***
		*Female*	83.3% (10)	82.1% (78)	82.1% (32)	
	*Specialty*	*Internal Medicine*	33.3% (4)	40.0% (38)	69.2% (27)	***0.005***
		*Family Medicine*	66.7% (8)	60.0% (57)	30.8% (12)	
	*Position*	*Resident*	75.0% (9)	83.2% (79)	66.7% (26)	*0.165*
		*Specialist*	25.0% (3)	11.6% (11)	20.5% (8)	
		*Consultant*	0.0% (0)	5.3% (5)	12.8% (5)	
	*Experience*	*<5 years*	33.3% (4)	60.0% (57)	53.9% (21)	*0.136*
		*5–10 years*	41.7% (5)	26.3% (25)	17.9% (7)	
		*>10 years*	25.0% (3)	13.7% (13)	28.2% (11)	
	*Training*	*No*	66.7% (8)	48.4% (46)	30.8% (12)	0.052
		*Yes*	33.3% (4)	51.6% (49)	69.2% (27)	
Practice	Total		13.7% (20)	40.4% (59)	45.9% (67)	
	*Sex*	*Male*	35.0% (7)	13.6% (8)	16.4% (11)	*0.088*
		*Female*	65.0% (13)	86.4% (51)	83.6% (56)	
	*Specialty*	*Internal Medicine*	60.0% (12)	49.2% (29)	41.8% (28)	*0.334*
		*Family Medicine*	40.0% (8)	50.9% (30)	58.2% (39)	
	*Position*	*Resident*	85.0% (17)	81.4% (48)	73.1% (49)	*0.712*
		*Specialist*	10.0% (2)	11.9% (7)	19.4% (13)	
		*Consultant*	5.0% (1)	6.8% (4)	7.5% (5)	
	*Experience*	*<5 years*	65.0% (13)	62.7% (37)	47.8% (32)	***0.008***
		*5–10 years*	30.0% (6)	28.8% (17)	20.9% (14)	
		*>10 years*	5.0% (1)	8.5% (5)	31.3% (21)	
	*Training*	*No*	70.0% (14)	42.4% (25)	40.3% (27)	0.055
		*Yes*	30.0% (6)	57.6% (34)	59.7% (40)	

Percentages are column-based within each domain. *p*-values represent comparisons across categories using Chi-square or Fisher’s exact test, as appropriate. Statistically significant *p*-values are bolded.

**Table 3:** Logistic Regression Analysis of Predictors of High Knowledge, High Attitude, and High Practice Levels (N = 146).

Predictor	Knowledge aOR (95% CI)	*p*-Value	Attitude aOR (95% CI)	*p*-Value	Practice aOR (95% CI)	*p*-Value
Sex (Male vs. Female)	0.91 (0.34–2.43)	0.85	1.18 (0.43–3.24)	0.741	1.41 (0.58–3.44)	0.445
Specialty (FM vs. IM)	**2.42 (1.03–5.72)**	**0.043**	**0.32 (0.14–0.76)**	**0.01**	1.75 (0.83–3.70)	0.141
Position (Senior vs. Resident)	1.81 (0.59–5.51)	0.299	1.49 (0.47–4.68)	0.497	1.75 (0.61–5.00)	0.296
Experience (≥5 vs. <5 years)	1.28 (0.50–3.28)	0.606	1.10 (0.42–2.86)	0.846	1.54 (0.68–3.46)	0.297
Training (Yes vs. No)	**7.23 (2.99–17.49)**	**<0.001**	2.16 (0.95–4.90)	0.066	1.48 (0.75–2.93)	0.254

A binary logistic regression model was used to estimate the odds of achieving high knowledge, high attitude, and high practice levels (≥80%) compared with low/moderate levels. Senior = Specialist + Consultant combined; experience categorized as <5 years vs. ≥5 years. aOR = adjusted odds ratio; CI = confidence interval. Bolded values indicate statistically significant associations at *p* < 0.05.

## Data Availability

The data supporting the findings of this study are available from the corresponding author upon reasonable request.
